# Anti-Inflammatory, Anti-Hyperglycemic, and Anti-Aging Activities of Aqueous and Methanolic Fractions Obtained from *Cucurbita ficifolia* Bouché Fruit Pulp and Peel Extracts

**DOI:** 10.3390/molecules30030557

**Published:** 2025-01-26

**Authors:** Tiago E. Coutinho, Carlos Martins-Gomes, Liliana Machado-Carvalho, Fernando M. Nunes, Amélia M. Silva

**Affiliations:** 1 Centre for Research and Technology of Agro-Environmental and Biological Sciences (CITAB), Cell Biology and Biochemistry Laboratory, University of Trás-os-Montes and Alto Douro (UTAD), Quinta de Prados, 5000-801 Vila Real, Portugal; tecoutinho@utad.pt (T.E.C.); camgomes@utad.pt (C.M.-G.); lilianac@utad.pt (L.M.-C.); 2 Chemistry Research Centre-Vila Real (CQ-VR), Food and Wine Chemistry Laboratory, University of Trás-os-Montes and Alto Douro (UTAD), Quinta de Prados, 5000-801 Vila Real, Portugal; fnunes@utad.pt; 3 Department of Biology and Environment, School of Life Sciences and Environment, University of Trás-os-Montes and Alto Douro (UTAD), 5000-801 Vila Real, Portugal; 4 Department of Chemistry, School of Life Sciences and Environment, University of Trás-os-Montes and Alto Douro (UTAD), 5000-801 Vila Real, Portugal; 5 Institute for Innovation, Capacity Building and Sustainability of Agri-Food Production (Inov4gro), University of Trás-os-Montes and Alto Douro (UTAD), Quinta de Prados, 5000-801 Vila Real, Portugal

**Keywords:** *Cucurbita ficifolia* Bouché, antioxidant, anti-inflammatory, neuroprotector, anti-aging, cell cycle arrest, functional foods, reactive oxygen species

## Abstract

The *Cucurbita* genus comprises various species that are globally consumed and that are commonly used for their nutritional value but also for medicinal applications. Within the *Cucurbita* genus can be found *Cucurbita ficifolia* Bouché, a species that is understudied regarding its potential value for the food industry, as a functional food, and for the pharmaceutical industry, as a source of nutraceuticals. Therefore, in this study we investigated the phytochemical composition and bioactivities of aqueous (AF) and methanolic (MF) fractions of *C. ficifolia* pulp and peel hydroethanolic (HE) extracts. HPLC-DAD-MS^n^ and HPAEC-PAD analyses of extracts’ fractions revealed a low content of polyphenols and a significant content of sugars. Through in vitro inhibition assays of the enzymes alpha-amylase, acetylcholinesterase (AChE), and elastase, all fractions showed, respectively, antidiabetic, neuroprotective, and anti-aging activities. The safety profile and anti-tumoral activities were evaluated in various cell models (Caco-2, HaCaT, HepG2, and RAW 264.7), and results showed that the fractions obtained from pulp extract induce no/low cytotoxicity, while the methanolic fraction of peel induced cytotoxicity in all cell lines. At non-cytotoxic concentrations, aqueous and methanolic fractions of both extracts significantly inhibited nitric oxide (NO) production in lipopolysaccharide (LPS)-stimulated RAW 264.7 cells, revealing anti-inflammatory activity. Flow cytometry analysis showed that both aqueous fractions increased basal levels of glutathione (GSH) in Caco-2 cells, while not inducing oxidative stress, revealing potential as antioxidant dietary agents. However, the MF of peel HE extract induced oxidative stress in Caco-2 cells, as it increased reactive oxygen species (ROS) and lipid peroxidation. AF fraction of peel extract induced cell cycle arrest in the G0/G1 phase, while the other fractions induced cell cycle arrest in the S phase. In conclusion, *Cucurbita ficifolia* fruit presents potential as a functional food but also as a potential source of nutraceuticals, and peel waste products can be valorized by pharmaceutical and cosmeceutical industries as sources of bioactive molecules.

## 1. Introduction

The most basic function of food intake is to meet the nutritional requirements to sustain life, providing essential macronutrients and micronutrients such as lipids, carbohydrates, proteins, minerals, and other classes of compounds. However, in addition to these compounds used to fulfill basic daily needs, most foods commonly found in the human diet are also a source of other additional compounds that may have health-promoting properties [1,2]. These food products, commonly defined as functional foods, provide health benefits beyond nutrition, thus reducing the risks of disease and/or alleviating some disease symptoms [3,4,5]. The concept of functional food was first coined in the 1980s and since then its definition has been under debate [6]; however, it is commonly accepted that, in addition to some conventional foods, functional foods also comprise a wide variety of other foods, including enriched, fortified, or enhanced foods as well as foods cleared from anti-nutritional compounds that provide health benefits [5,6]. Plant products emerge as the main source of molecules with health-promoting properties; examples of these molecules include polyphenols, saponins, carotenes, polyunsaturated fatty acids, or alkaloids [7]. A wide range of bioactivities have been reported for functional foods and their components, including anti-cancer, anti-diabetic, neuroprotective, antimicrobial, and antioxidant activities, and others [2,7].

Among the various plant products with high representation in the human diet, we can find foods from species of the *Cucurbita* L. genus, which have a significant market value, and whose best-known fruits are pumpkins and courgettes [8]. This genus comprises 14 species, among which *Cucurbita moschata* Duchesne (butternut squash), *Cucurbita maxima* Duchesne (Hubbard squash), *Cucurbita argyrosperma* K. Koch (cushaw pumpkin or silver-seed gourd), *Cucurbita pepo* L. (summer squash, pumpkin), and *Cucurbita ficifolia* Bouché (white pumpkin, chila pumpkin, ‘chilacayote’) can be found in human diets, and, apart from the pulp of the fruit, which is the most consumed portion, the seeds, shoots, and flowers are also consumed [8,9,10,11]. In addition to the nutritional value, other properties have been reported for the *Cucurbita* genus, supporting their use in traditional medicine, based on health-promoting activities such as antioxidant, antidiabetic, anti-cancer, antifungal, anti-parasitic, immunomodulatory, hepatoprotective, or for their benefits to the cardiovascular system [9,11,12,13,14,15].

*Cucurbita ficifolia* is commonly consumed in several countries. Its fruit is used as a vegetable in soups or to produce jams, candies, and sweets [16,17], and also fermented soft alcoholic beverages [10]. The edible seeds can be consumed raw or roasted or can be used to obtain seed oil [10]. Leaves, flowers, and shoots can be used in salads, soups, among other dishes [18,19]. The traditional cultivation and culinary use of *C. ficifolia* is well described in Central and South America, Africa, Asia, and Europe [18]. In traditional medicine, *C. ficifolia* is used for the treatment of type 2 diabetes mellitus [19], but other benefits for human health have been described, such as antioxidant, anti-inflammatory, and antihypertensive, and it is also responsible for increasing immunity and fertility [20].

Regarding the scientific validation of *C. ficifolia* bioactivities, it was shown that an aqueous extract of *C. ficifolia* fruit (200 mg/kg) reduced inflammatory markers (TNF-α and IL-6) in obese mice with systemic chronic inflammation (obesity model induced by monosodium glutamate) and decreased body mass [21]. In another study, using diabetes rats, induced with streptozotocin, it was reported that the administration of an aqueous extract of *C. ficifolia* fruit (200 mg/kg) increased glucose tolerance, hepatic glycogen levels, and plasma insulin levels and decreased glycolyzed hemoglobin levels [22].

Some phytoconstituents have already been identified in other *Cucurbita* spp., and it was reported that polysaccharides can comprise up to 60% of their composition, among which galactans, glucans, galactoglucans, and galactomannans, for example, were identified [23]. These polysaccharides present several health-promoting properties and, depending on their structure, may induce antidiabetic, antioxidant, and antitumor immunological activities [23,24]. In general, *Cucurbita* spp. fruits present low fat levels; species with colored fruits usually present high content in carotenoids, and various phenolic compounds have been identified, namely *p*-coumaric acid, protocatechuic acid, salicylic acid, chlorogenic acid, hesperidin, and eriodictyol derivatives [9,25]. In other studies, these phenolic compounds have been described to exert several biological activities, from antioxidant to anti-cancer, anti-aging, anti-inflammatory, and others [26].

Regarding *C. ficifolia* phytoconstituents, compounds such as D-chiro inositol, phenolic acids, and flavonoids have been identified [17]. Nevertheless, *C. ficifolia* fruits’ phytochemical composition is still understudied. Even more, most studies address the compositions and bioactivities of the fruit’s pulp and seeds but do not consider the potential of the fruit’s peel as a source of nutraceuticals. As only the pulp and seeds of pumpkins are traditionally consumed, the peel can be considered as a by-product. In other *Cucurbita* spp., the peel is a source of beneficial bioactive compounds, with various applications in the food, pharmaceutical, and cosmetic industries [27].

Also, the potential of *C. ficifolia* fruit as a functional food and the valorization of its by-products’ phytoconstituents using cellular and enzymatic models must be further addressed. These would provide a deeper scientific validation for the health-promoting effects reported to the fruit but also would valorize its by-products, fostering a circular economy. Given that, to date, only a small number of studies have been published on the bioactivities of *C. ficifolia*, we understand that before health-promoting effects in animal models are studied, initial screening must be performed in human cell models. Since these fruits are ingested, special attention should be paid to cellular models originating from gastrointestinal tract tissues and liver tissues, the former being related to digestion and absorption and the latter being related to the first-pass effect.

Thus, the main objective of this work was to evaluate the phytochemical composition of *C. ficifolia* fruit’s peel and pulp extracts and then to evaluate their potential neuroprotective, anti-diabetic, and anti-aging activities using enzymatic assays; and, by using human cell lines, it is also an objective to evaluate the cytotoxic effect of these extracts and their anti-proliferative potential. Particular emphasis will be given to the extracts’ effect on cellular oxidative stress, genotoxicity, cell cycle progression, and their capacity to inhibit inflammation in an LPS-stimulated macrophage cell model.

## 2. Results and Discussion

### 2.1. Extraction and Fractionation Yields, Chemical Composition, and Antioxidant Activity

In this work, an exhaustive hydroethanolic extraction method was performed aiming to obtain all extractable polyphenols present in the pulp and peel of *C. ficifolia* fruit. An extraction yield of 59.91 ± 5.22% and 28.66 ± 1.03% was obtained for pulp and peel, respectively (Table 1). As reported for other food matrices [28], the extraction method used allows the extraction of 99% of all polyphenols present in the plant material, considering three sequential extraction steps. However, as described in Section 3.2.1, exhaustive hydroethanolic extraction was performed considering four sequential extraction steps to assure the extraction of all polyphenols (≥99%). Xia and Wang [29] carried out a methanolic extraction (70% *v*/*v*), at 50 °C for 1 h of *C. ficifolia* fruit pulp, obtaining a yield of 7.2% and thus much lower than the extraction yields reported in this work (Table 1). Akhter et al. [30] reported yields of ~26% and ~29% for aqueous extracts of the *Cucurbita moschata* fruit pulp and peel, respectively, which can be compared to the yield obtained for the Peel HE extract (~29%) obtained in this work (Table 1).

Pulp and peel HE extracts were then fractionated, resulting in an aqueous fraction (AF) and a methanolic fraction (MF) for each HE extract. The yield of these fractions is presented in Table 1. As observed, for each HE extract, the yield of the respective AF is significantly higher than the yield of the MF (*p* < 0.05). Comparing fraction yields of pulp and peel, higher values were obtained for Peel HE fractions. To the best of our knowledge, this is the first work reporting the fractionation of *C. ficifolia* fruit extracts.

An initial assessment of the chemical composition was determined by colorimetric assay. The content in total phenols (TPC), *ortho*-diphenols (ODC), and flavonoids (TFC) is presented in Table 1. Significant differences were not observed between the pulp HE fractions (Pulp HE-AF vs. Pulp HE-MF; *p* > 0.05), and when comparing pulp fractions to Peel HE-AF. However, Peel HE-MF TPC is significantly higher (1.70 to 1.93 times) than the other fractions (*p* > 0.05). A similar pattern is observed for ODC, where Peel HE-MF presents the highest content in *ortho*-diphenols, with a content 1.58, 1.55, and 1.71 times higher than Pulp HE-AF, Pulp HE-MF, and Peel HE-AF, respectively (Table 1).

Regarding TFC, no significant differences were observed between AFs or between MFs of pulp HE and peel HE (Table 1). However, both MFs presented higher TFC than AFs (*p* < 0.05). Stryjecka et al. [31] evaluated TPC and TFC by colorimetric methods in hidromethanolic extracts from the pulp of *C. ficifolia* fruit, reporting TPC and TFC values of 0.34 mg GAE/g FW and 0.01 mg quercetin equivalents/g FW, respectively. To the best of our knowledge, this is the first work reporting TPC and TFC values in fractions obtained from the HE extracts from the peel and pulp of *C. ficifolia* fruits. However, Gaweł-Bęben et al. [32] reported TPC and TFC values, using colorimetric methods, in ethanolic extracts obtained from peels of other *Cucurbita* spp. (e.g., *C. maxima* and *C. moschata*). Considering the fraction yield, the peel HE extract of *C. ficifolia* (AF plus MF) presented a TPC content of ~58 mg GA eq./g extract and a TFC content of ~114 mg/GA eq./g extract (Table 1), both higher than the values reported for TPC and TFC of ethanolic extracts obtained from the peel of other *Cucurbita* spp. (TPC values between 4.62 and 17.60 mg GA eq./g dw; TFC values between 1.79 and 3.60 mg quercetin eq./g dw [32]).

Concerning antioxidant activity (Table 1), all fractions were able to scavenge ABTS cation radical (ABTS^•+^). Comparing fractions, MFs showed higher ABTS^•+^ scavenging capacity than the AFs, and the peel HE fractions showed higher scavenging activity than the equivalent pulp HE fractions (Table 1). The Peel HE-MF was the fraction producing the highest ABTS^•+^ scavenging (0.66 ± 0.05 mmol Trolox eq/g extract; Table 1), correlating with the highest TPC and TFC. Gaweł-Bęben et al. [32] reported the ABTS^•+^ scavenging activity of various aqueous and ethanolic extracts of *Cucurbita* spp. fruit peels (e.g., *C. maxima* and *C. moschata*), showing values ranging between 0.003 and 0.004 mmol Trolox eq/g extract, and thus lower than the scavenging activity here reported for fractions of *C. ficifolia* fruit HE extracts (Table 1).

Also concerning antioxidant activity, in Table 1 it is shown that AF and MF from both Pulp HE and Peel HE extracts inhibited hydroxyl radical production. A higher inhibition was observed for both MFs (45%) when compared to AFs (34% for pulp and 28% for peel) (*p* < 0.05). There were no significant differences between MFs, while Pulp HE-AF presented a significantly higher inhibition than Peel HE-AF. To the best of our knowledge, this is the first report of hydroxyl radical scavenging by extracts of *Cucurbita* spp.

### 2.2. Phytochemical Composition of Cucurbita ficifolia Bouché Extract Fractions

**Figure 1:**
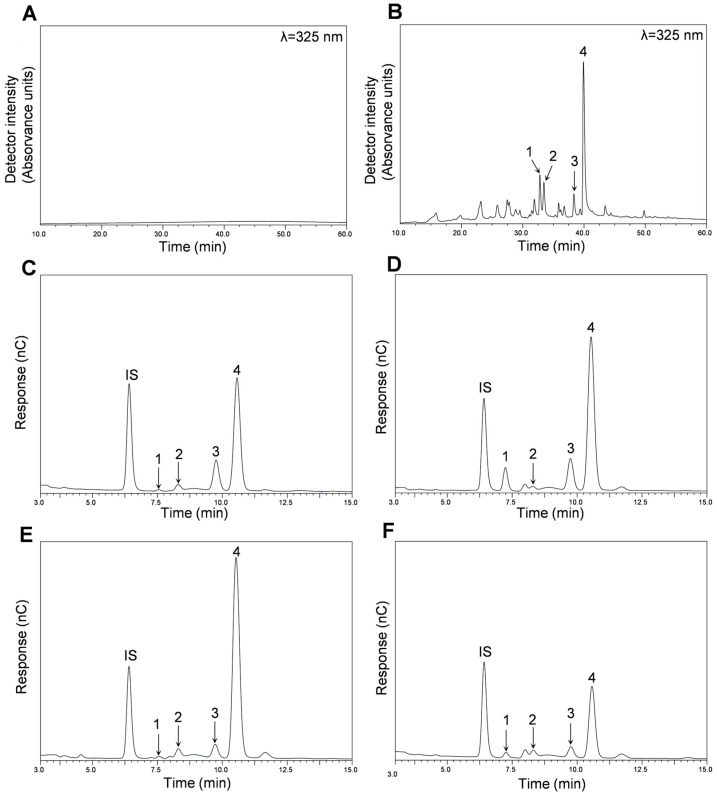
Composition of the different fractions (AF and MF) obtained from *C. ficifolia* fruit pulp and peel HE extracts. HPLC-DAD chromatograms obtained for Peel HE-AF (**A**) and Peel HE-MF (**B**). HPAEC-PAD chromatograms were obtained for Peel HE-AF (**C**), Peel HE-MF (**D**), Pulp HE-AF (**E**), and Pulp HE-MF (**F**). In panel (**B**): **1**, (Iso)rhamnetin-(?)-*O*-deoxy-hexose-hexose-(?)-*O*-deoxy-hexose; **2**, Quercetin-(?)-*O*-deoxy-hexose-hexose; **3**, Luteolin-(?)-*O*-deoxy-hexose-hexose; **4**, (Iso)rhamnetin-(?)-*O*-deoxy-hexose-hexose. In panels (**C**–**F**): **IS**, internal standard; **1**, rhamnose; **2**, arabinose; **3**, galactose; **4**, glucose.

The main polyphenols of the different fractions (AF and MF) obtained from Pulp HE and Peel HE were identified and quantified using HPLC-DAD-ESI-MS^n^ from chromatograms such as those presented in Figure 1 (Peel-HE AF: A and Peel-HE MF: B). Identification and quantification of simple sugars were performed by HPAEC-PAD from chromatograms as those presented in Figure 1C–F.

As seen in Figure 1A, no polyphenols were detected in Peel HE-AF (considering the chromatographic conditions used; (Section 3.5 for methods). HPLC-DAD-ESI/MS^n^ analysis of both Pulp-HE fractions also confirmed the absence of polyphenolic compounds (Table 2). Nonetheless, four flavonoid glycoside derivatives were identified and quantified in Peel HE-MF, as shown in Table 2 and as shown in Figure S1 (Supplementary Material).

As seen in Table 2, three quercetin derivatives were identified, among which two O-methylated derivatives and all derivatives containing sugar moieties (Table 2). Compound 1 was identified as (iso)rhamnetin-(?)-*O*-deoxy-hexose-hexose-(?)-*O*-deoxy-hexose due to the presence of a pseudo-molecular ion with *m*/*z* of 769, a fragmented ion with *m*/*z* 623, implying the loss of a 146 Da fragment, which corresponds to a deoxyhexose, and also a fragment with *m*/*z* 315, that is due to the loss of a 308 Da fragment, indicating the loss of a hexose (162 Da) and deoxyhexose (146 Da) residues. The fragment with *m*/*z* 315 corresponds to the aglycone, which can be either rhamnetin or isorhamnetin linked to the same position of the aglycone, showing a fragmentation pattern already described by Cvetković et al. [33]. Compound 2 (Table 2) was identified as quercetin-(?)-*O*-deoxy-hexose-hexose, presenting a fragmentation pattern similar to compound 1, with a loss of 308 Da from the pseudo-molecular ion with an *m*/*z* of 609 to the fragment with *m*/*z* of 301, which corresponds to the loss of deoxy-hexose-hexose, and being the fragment with *m*/*z* of 301 correspondent to quercetin aglycone. This fragmentation pattern was previously described by Abraão et al. [34]. Compound 4 was identified as (iso)rhamnetin-(?)-*O*-deoxy-hexose-hexose, and its fragmentation pattern is identical to that of compound 1, but showing a pseudo-molecular ion with an *m*/*z* of 623, and thus presenting the absence of a deoxy-hexose moiety when compared to compound 1. Additionally, MS^2^ analysis revealed the presence of the fragment with *m*/*z* of 300, which is the loss of a methyl group, as rhamnetin or isorhamnetin are *O*-methylated derivatives of quercetin. This fragmentation pattern was previously described [35,36].

In addition to quercetin derivatives, a luteolin derivative was identified, namely luteolin-(?)-*O*-deoxy-hexose-hexose (Compound 3). The pseudo-molecular ion presents an *m*/*z* of 593, and the presence of a fragment with 285 Da was detected, corresponding to luteolin aglycone, and once again indicating the loss of a deoxy-hexose-hexose moiety (308 Da). This fragmentation pattern was previously reported by other authors (Mona M. Marzouk and [37]). Thus, all glycoside derivatives identified in the fractions obtained from *C. ficifolia* fruit HE extracts reported in this study (Table 2) present the moiety deoxy-hexose-hexose. Mona M. Marzouk and [37] observed the presence of (iso)rhamnetin-(?)-*O*-deoxy-hexose-hexose-(?)-*O*-deoxy-hexose and of (iso)rhamnetin-(?)-*O*-deoxy-hexose-hexose in ethanolic extracts obtained from the pulp of *C. pepo*. Luteolin-(?)-*O*-deoxy-hexose-hexose was previously identified in extracts of *C. moschata* [38]. Mansour et al. [39] reported the presence of quercetin-(?)-*O*-deoxy-hexose-hexose in extracts obtained from the peel of *C. maxima* fruit.

Comparing the results of colorimetric methods with those obtained by HPLC, we verified that TPC quantified by colorimetric methods (Table 1) is higher than that quantified by HPLC (Table 2). The method used for colorimetric quantification of TPC is based on Folin–Ciocalteau reagent, which is known to react with various reducing compounds other than phenolics, namely amino acids, proteins, sugars, or vitamin derivatives, leading to an overestimation of the TPC [40]. Thus, while the Folin–Ciocalteau method is suitable for samples such as extracts obtained from aromatic plants [40], which are rich in phenolic compounds, it is not suitable for samples with low contents of phenolics. However, although the fractions obtained from *C. ficifolia* HE extracts reported in the present study are not rich in phenolic compounds, they may contain other molecules such as proteins, carbohydrates, fibers, minerals, vitamins, and amino acids, among others [41], with reducing and with relevant bioactive properties.

In Table 3, the quantification of simple sugars after acid hydrolysis by HPAEC-PAD in AF and MF fractions of pulp and peel HE extracts is presented.

In all fractions, the presence of 4 sugars was observed, namely rhamnose, arabinose, galactose, and glucose (Table 3). Glucose is the sugar present in higher content in all fractions, followed by galactose. Comparing fractions, a significantly higher content was observed for Pulp HE-AF when compared to Pulp HE-MF (*p* < 0.05), while Peel-AF presented on average a lower content in glucose when compared to Peel-MF, but the difference is not significant (*p* > 0.05). The highest glucose content was observed for Pulp-AF.

Concerning galactose content (Table 3), a higher content was observed in peel HE fractions when compared to pulp HE fractions, and no statistically significant differences between AF and MF, within each extract, were observed. A higher arabinose content was observed in the MF fractions, regardless of the extract, although in general MFs have low contents of this monosaccharide.

Regarding rhamnose content, both MF fractions have significantly higher content than AF fractions. Nevertheless, Peel HE-MF content in rhamnose is 4.18 times higher than that observed for Pulp HE-MF (Table 2). The total sugar content represents 25% and 11% of the total content of Pulp HE-AF and Pulp HE-MF, respectively, and approximately 18% and 23% of AF and MF of Peel HE, respectively.

Among the monosaccharides identified, these may be constituents of some neutral polysaccharides already identified in other species of Cucurbita, such as homopolysaccharides (galactans, glucans) and heteropolysaccharides (galactoglucans), mostly containing galactose and glucose. This type of polysaccharide has been described to present numerous activities, such as antioxidant, anti-diabetic, anti-tumor, regulation of the immune system, among others [23].

### 2.3. Extracts Capacity to Inhibit Metabolically Relevant Enzymes

To evaluate the potential biological effects of these extracts, the ability of these to inhibit the activity of metabolically relevant enzymes was further evaluated. Table 4 presents the results of the anti-enzymatic activity induced by the fractions obtained from *C. ficifolia* fruit extracts, aiming to evaluate their potential effects, such as antidiabetic (α-amylase and α-glucosidase), neuroprotective (AChE and tyrosinase), and anti-aging (elastase and tyrosinase), as was reported for other extracts [42].

In terms of neuroprotective activity, we observed that all fractions significantly inhibited acetylcholinesterase (AChE) activity, with the inhibition values between 21% and 23%, regardless of the fraction type or source (*p* > 0.05). Considering other *Cucurbita* spp., *C. maxima* ethanolic and hexane extracts also demonstrated the ability to inhibit AChE activity, with inhibitory activity between 40% and 50%, when tested at 0.25 mg/mL [43], and thus presenting higher AChE inhibitory potential than the fractions obtained from *C. ficifolia* fruit extracts here reported (Table 4).

Regarding the inhibition of tyrosinase, it was observed that both fractions (AF and MF) obtained from Peel-HE and Pulp-HE extracts inhibited the activity of this enzyme. The highest inhibition was observed for Pulp HE-MF, which inhibited 12% of tyrosinase activity (Table 4). On average, both MF fractions produced higher enzymatic inhibition than AF (Table 4). Tyrosinase is responsible for the synthesis of melanin in skin and hair cells, and, in the brain, this enzyme has been implicated in the formation of neuromelanin, which, by interaction with toxicants, has been attributed a role in the origin of several neurodegenerative diseases [44]. Therefore, we can conclude that the effect of these fractions as a skin lightener, as well as their neuroprotective potential through tyrosinase inhibition, is low.

The highest anti-enzymatic activity of these fractions was observed in the anti-elastase assay. Both fractions obtained from Pulp-HE completely inhibited elastase activity (100% inhibition) at 300 µg/mL. Peel HE-MF inhibited 96% of enzyme activity, showing an anti-elastase potential similar to the Pulp HE fractions (Table 4). On the other hand, Peel HE-AF produced the lowest inhibition (73%), being significantly different from Peel HE-MF (*p* < 0.05), although still presenting a high potential as an anti-elastase agent. To the best of our knowledge, this is the first work reporting the anti-elastase activity of *C. ficifolia* fruit extracts. Since elastase is one of the metalloproteinases involved in the breakdown of extracellular matrix protein (e.g., elastin) breakdown a process that is implicated in wrinkle formation and loss of skin firmness [45], inhibitors of this enzyme may have an anti-aging effect. Thus, considering the results here presented, it is worth highlighting that these extracts can be a source of valuable bioactive compounds for the cosmetic industry.

Regarding the therapeutic targets to achieve a reduction in hyperglycemia, there are several strategies [46], including the inhibition of enzymes involved in polysaccharide and oligosaccharide hydrolysis, namely by natural products, under constant study. Reduction in monosaccharide bioavailability not only controls hyperglycemia but also controls obesity and metabolic syndrome [46]. Thus, in this work, it was observed that all fractions significantly inhibited α-amylase activity, with the MFs having a better inhibitory effect than AFs, regardless of the extract (Table 4). The highest inhibition was observed for Pulp HE-MF (35%), this value being significantly higher than the others (*p* < 0.05). No significant differences were observed between the inhibition produced by the two AFs (*p* > 0.05). On the other hand, none of the extract fractions was able to inhibit α-glucosidase at 300 µg/mL (Table 4). Thus, MF fractions have higher potential as anti-hyperglycemic agents through the inhibition of α-amylase. Several studies on animal models of diabetes show a decrease in blood glucose levels when *C. ficifolia* is added to the diet [16,17], correlating the reduction in blood glucose levels with the inhibition of the α-amylase, a mechanism that involves inhibiting the breakdown of starch into simple sugars, thus reducing their absorption [47]. Thanh et al. [48] also showed that a polysaccharide component of *Cucurbita pepo* was able to inhibit α-amylase activity evaluated using an in vitro assay. Other parts of the pumpkin plant have been shown to have anti-α-amylase activity; for example, a methanolic extract of *C. maxima* leaves inhibited α-amylase activity with an IC**_50_** value of 2.1 mg/mL (comparing with acarbose 0.62 mg/mL) [49]. In another study, *C. maxima* seeds extract inhibited α-amylase activity with an IC**_50_** value of 138 μg/mL, but also inhibited α-glucosidase (IC_50_ = 20 μg/mL) and dipeptidyl peptidase IV (IC_50_ = 246 μg/mL) [50], showing that seeds have a high anti-hyperglycemic effect. Knowing the potential anti-hyperglycemic effect of food products is crucial to reduce the incidence of diabetes and also to manage glycemia in diabetic and prediabetic patients.

### 2.4. Assessment of the Safety Profile of AF and MF Fractions Obtained from Pulp and Peel HE Extracts

#### 2.4.1. Safety Profile of Pulp HE Extracts Fractions

**Figure 2:**
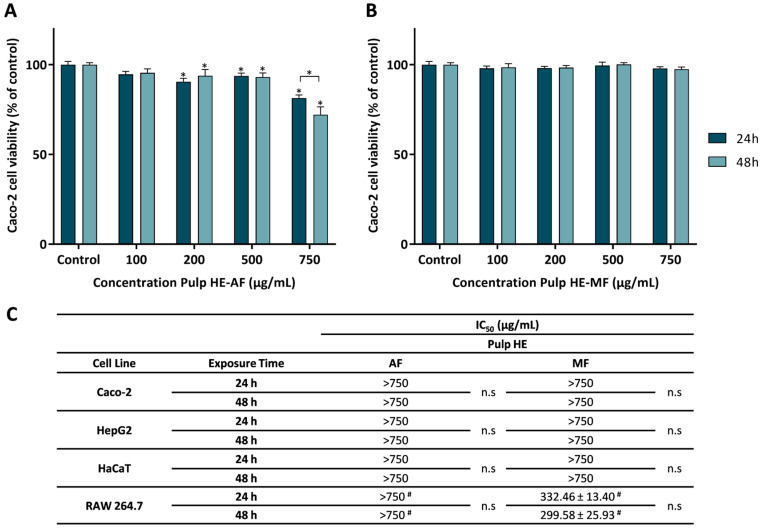
Effect of the aqueous fraction (AF) (**A**) and the methanolic fraction (MF) (**B**) of the hydroethanolic extract of the pulp (Pulp HE) on Caco-2 cells after 24 h and 48 h of exposure. (**C**) The IC_50_ values were calculated from experiments as that depicted in A and B for Caco-2, HepG2, HaCaT, and RAW 264.7 cells exposed to both fractions (AF and MF). In panels A and B, statistically significant differences (*p* < 0.05) between test concentrations and respective controls are denoted by an asterisk (*), and differences between exposure times are denoted by an * over a square bracket; in panel C, (#) denotes differences between fractions, and n.s. denotes non-significant. Results are expressed as (mean ± SD, *n* = 4).

Aiming to evaluate the safety profile of the different fractions (AF and MF) of Pulp HE, four different cell lines were used (Caco-2: human colorectal adenocarcinoma, HaCaT: human keratinocytes, HepG2: human hepatocarcinoma, and RAW 264.7: mouse macrophages derived from Abelson murine leukemia virus-induced tumor). Cells were exposed to AF and MF at concentrations ranging between 0 and 750 µg/mL for 24 or 48 h (as detailed in the Section 3). Figure 2 shows the obtained results.

Figure 2A shows that Caco-2 cells exposed to Pulp HE-AF at concentrations up to 500 μg/mL have a viability above 90%. Being the exposure to 100 μg/mL not statistically different from control (*p* < 0.05). In addition, cells exposed to concentrations ≥ 200 μg/mL of Pulp HE-AF show a statistically significant reduction in viability in a dose-dependent effect. Concerning the exposure time effect, only for the highest concentration (750 μg/mL), a significant decrease in cell viability (*p* < 0.05) was observed, with the cell viability values of 81.45 ± 1.72% and 72.17 ± 4.33% at 24 and 48 h exposure, respectively.

As observed in Figure 2B, Pulp HE-MF, up to 750 μg/mL, did not change Caco-2 cell viability, regardless of the exposure time, when compared to the control (*p* > 0.05).

Wu et al. [51] reported that a refined polysaccharide fraction of pumpkin (species not specified) did not induce cytotoxicity in Caco-2 cells at concentrations up to 800 µg/mL, and thus in line with the results here presented for Pulp HE-MF.

From dose-response curves, as those presented in Figure 2A,B, obtained by exposing other cell lines to the AF and MF Pulp-HE extract fractions, the IC_50_ values were calculated and are presented in Figure 2C. As in Caco-2 cells, HepG2 and HaCaT cells exposed to either fraction (at concentrations up to 750 µg/mL) did not reduce cell viability below 50% (Figure 2C), and the viability pattern was identical to that of Caco-2 for exposure times of 24 and 48 h. However, RAW 264.7 cells show higher sensitivity to Pulp HE-MF extract compared to the other cell lines, since the calculated IC_50_ values are 332.46 µg/mL and 299.58 µg/mL for 24 h and 48 h exposure, respectively. Although on average there is a time-dependent effect, differences are not statistically different (Figure 2C). Also, comparing AF vs. MF, RAW 264.7 cell viability when cells were exposed to AF at 750 µg/mL was higher than 50% (of control).

Shen et al. [52] showed that 100 μg/mL of polysaccharides extracted and purified from pumpkin pulp (unspecified species) induce cytotoxicity to HepG2 cells exposed for 24 h (75% cell viability), and thus present higher cytotoxicity than the fractions studied in the present research (Figure 2C). This could be due to the high concentration of polysaccharides in the fractions obtained by Shen et al. [52]. To the best of our knowledge, this is the first report on the safety profile of HE extracts from *Cucurbita* spp. pulp in HaCaT cells.

#### 2.4.2. Safety Profile of Peel HE Extracts Fractions

**Figure 3:**
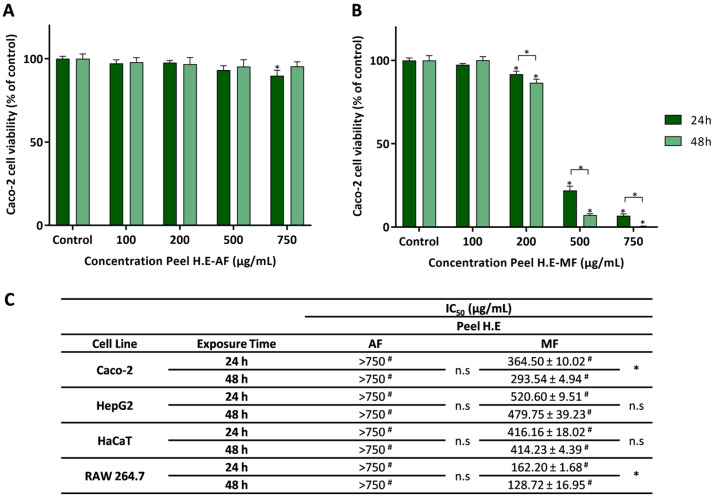
Effect of the aqueous fraction (AF) (**A**) and methanolic fraction (MF) (**B**) of *C. ficifolia* peel hydroethanolic extract (Peel HE) on Caco-2 cell lines after 24 h and 48 h of exposure. (**C**) The IC_50_ values were calculated from experiments as that depicted in A and B, for Caco-2, HepG2, HaCaT, and RAW 264.7 cells exposed to both fractions (AF and MF). In panels A and B statistically significant differences (*p* < 0.05) between test concentrations and respective controls are denoted by an asterisk (*), and between exposure times by an * over a square bracket; in panel C, (#) denotes differences between fractions, and n.s. denotes non-significant. Results are expressed as (mean ± SD, *n* = 4).

The safety profile of the fractions, AF and MF, obtained from Peel HE extract was also evaluated in the four different cell lines (Caco-2, HaCaT, HepG2, and Raw 264.7) at different concentrations up to 750 µg/mL, with exposure for 24 or 48 h. Figure 3A shows the effect of AF on Caco-2 cell viability, with it being observed that the action of the extract at any concentration is practically null. As observed in Figure 3A, Peel HE-AF induced no/low cytotoxicity in Caco-2 cells, being only observed a slight decrease in viability for cells exposed to 750 μg/mL of Peel HE-AF for 24 h (*p* < 0.05).

Caco-2 cells exposed to Peel HE-MF (Figure 3B) present a significant decrease in cell viability at concentrations ≥ 200 μg/mL (*p* < 0.05). Additionally, an exposure time-dependent decrease in cell viability was observed at these concentrations. As observed, Peel HE-MF is the most cytotoxic of all fractions (Figure 2 and Figure 3). Considering the effect on Caco-2 cells, while for *C. ficifolia* pulp HE extract the AF is the one that produces higher cytotoxicity, for peel HE extract the MF induces higher cytotoxicity, being this correlated with the amount of phytochemicals (Table 2). To the best of our knowledge, this is the first report on the safety profile of fractions obtained from *Cucurbita* spp. fruit peel HE extracts on intestinal cell lines.

Figure 3C presents the IC_50_ values for the four cell lines exposed to Peel HE fractions, which were calculated from dose-response curves as those presented in Figure 1A,B. As observed, at the highest concentration (750 μg/mL), Peel HE-AF did not reduce cell viability below 50%, which was also observed for Pulp HE-AF (Figure 2C). On the other hand, Peel HE-MF (Figure 3C) produces higher cytotoxicity, with the macrophage cell line (RAW 264.7) being the one presenting the highest sensitivity. The order of toxicity of Peel HE-MF to the studied cell lines, measured with the increasing IC_50_ values, is as follows: RAW 264.7 < Caco-2 < HaCaT < HepG2, at both exposure times (Figure 3C).

HepG2 cells are used as a hepatocyte model, as these cells maintain standard hepatocyte membrane receptors and xenobiotic metabolization/detoxification enzymes. Also, it was previously reported that HepG2 cells have higher resistance to various natural products when compared to Caco-2 cells [42,53]. For Caco-2 and RAW 264.7 cells exposed to Peel HE-MF, a significant (*p* < 0.05) time-dependent effect was observed (Figure 3C).

Gaweł-Bęben et al. [32] reported the anti-proliferative activity of ethanolic and aqueous extracts obtained from the peel of *C. maxima* and *C. moschata* fruits. HaCaT cells exposed to the aqueous and ethanolic extracts obtained from the fruit peel of two cultivars of *C. maxima* and one cultivar of *C. moschata*, at concentrations ≥1000 μg/mL, showed a significant reduction in cell viability [32]. Overall, no/low cytotoxicity was observed in HaCaT cells exposed to aqueous and ethanolic extracts at concentrations up to 500 μg/mL [32], which agrees with the results here presented for the Pulp HE-AF and Pell HE-AF (Figure 2 and Figure 3). Zhang et al. [54], using an aqueous extract of the *C. moschata* fruit peel, reported no cytotoxic effect on HepG2 cells at concentrations up to 200 µg/mL, and thus in line with the results here reported for AF obtained from *C. ficifolia* fruit peel (Figure 3). Huang et al. [55] evaluated the cytotoxicity induced by a polysaccharide fraction obtained from *C. moschata* peel in RAW 264.7 cells, reporting no cytotoxicity at concentrations up to 200 µg/mL, but reduction in cell viability was observed in cells exposed to 500 μg/mL of extract. Some polysaccharides identified in pumpkin extracts present anti-tumoral activity, namely, apoptosis triggering [23].

### 2.5. Evaluation of Oxidative Stress Markers and Cell Cycle Arrest

Aiming to understand the mechanism behind the anti-proliferative effect observed for AF and MF fractions of *C. ficifolia* fruit peel and pulp HE extract, Caco-2 cells were used to study the modulation of intracellular ROS content, GSH content, lipid peroxidation, and cell cycle progression.

#### 2.5.1. Effect of Pulp HE Extract Fractions

**Figure 4:**
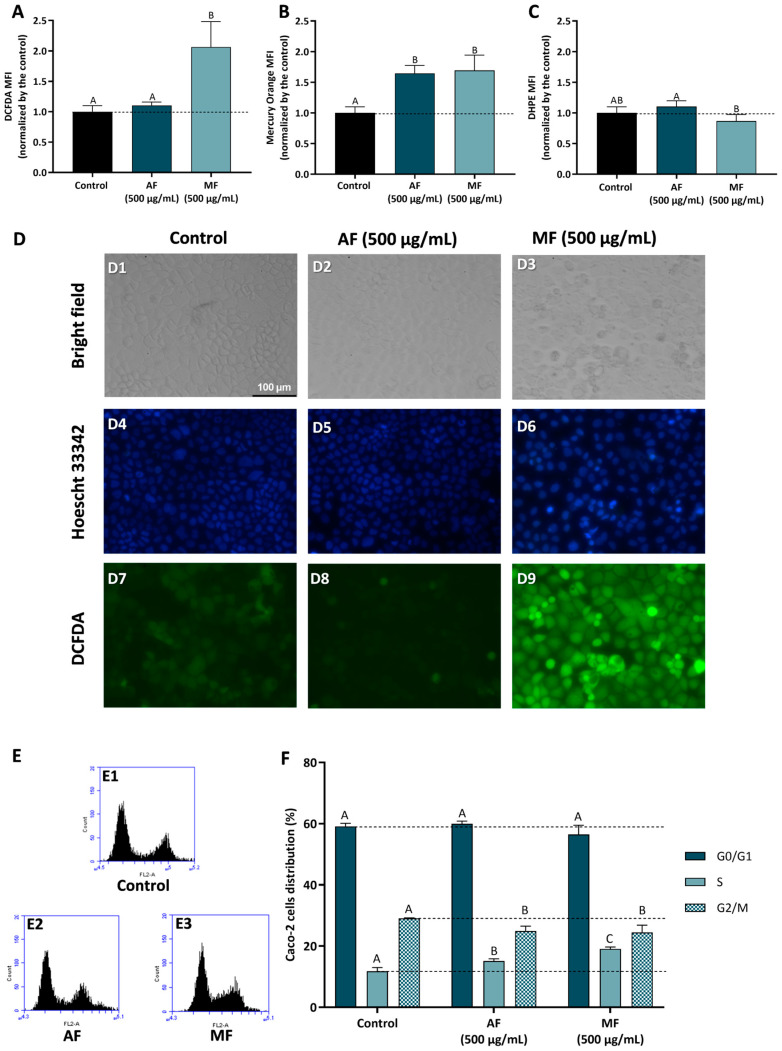
Effect of fractions obtained from *C. ficifolia* fruit pulp HE extract on oxidative stress markers and cell cycle arrest evaluated in Caco-2 cells exposed to 500 µg/mL of AF and MF (24 h exposure). (**A**–**C**): Intracellular reactive oxygen species (ROS) levels (evaluated as DCFDA MFI; panel (**A**)), glutathione content (GSH, evaluated as Mercury orange MFI, panel (**B**)), and lipidic peroxidation (evaluated as DHPE MFI, panel (**C**)). Panel (**D**): Analysis of Caco-2 cells’ morphology (bright-field), DNA integrity (Hoescht 33342; DAPI filter), and ROS content (DCFDA; FITC filter); Control (D1, D4, and D7), Peel HE-AF (D2, D5, and D8), and Pulp HE-MF (D3, D6, and D9); scale bar: 100 µm, magnification of 200×; (**E**,**F**): Assessment of fractions-induced cell cycle arrest (**F**) calculated from flow cytometry plots as exemplified in panel (**E**). Statistically significant differences (*p* < 0.05) between fractions are denoted with different letters. Results are presented as mean ± SD (*n* = 3). MFI: mean fluorescence intensity.

Figure 4 presents the results obtained in Caco-2 cells treated with 500 µg/mL of Pulp HE-AF and Pulp HE-MF fractions for 24 h, as denoted, regarding the modulation of oxidative stress markers and cell cycle progression. Cells exposed to Pulp-HE MF present an increase in oxidative stress, observed as a significant increase in intracellular ROS (*p* < 0.05; Figure 4A) and an increase in lipid peroxidation (*p* > 0.05; Figure 4C; denoted as a decrease in DHPE-FICT fluorescence, as the probe fluorescence is inversely correlated to lipid peroxidation). Pulp-HE MF also induced an increase in GSH content (Figure 4B), likely as a response to the oxidative stress, as GSH is a major endogenous non-enzymatic antioxidant [42]. On the other hand, the Pulp HE-AF did not increase ROS content or induce lipid peroxidation), it also induced an increase in GSH content. Thus, although Pulp HE-AF induced some cytotoxicity in Caco-2 cells when assessed by the Alamar Blue assay (Figure 2), it did not produce oxidative stress. On the other hand, we observed that only the MF fraction (which did not reduce cell viability) induces oxidative stress, meaning that these ROS levels are not sufficient to reduce cell viability.

Results obtained by flow cytometry were confirmed by bright field and fluorescence microscopy, as cells exposed to Pulp HE-AF present control-like morphology (Figure 4, panel D2), DNA integrity (Figure 4, panel D5), and lower ROS content similar to control (Figure 4, panel D8). Also supporting flow cytometry results, it is possible to observe an increase in DCF staining in Caco-2 cells treated with 500 µg/mL of Pulp HE-MF (Figure 4, panel D9) when compared to the control (non-exposed cells; Figure 4, panel D7) and Pulp HE-AF (Figure 4, panel D8). In addition, Caco-2 cells exposed to Pulp HE-MF present an increased percentage of cells with morphological changes and increased DNA staining, indicating DNA fragmentation.

It was also found that aqueous extracts of *C. ficifolia* pulp replenish GSH levels in liver, pancreas, and heart rat cells in a model of diabetes induced with streptozotocin (STZ) compared to healthy rats. The same happens for lipid peroxidation in the plasma of diabetic rats [22]. Thus, allowing us to understand that aqueous pulp extracts have the ability to maintain normal levels of ROS, GSH, and lipid peroxidation. Yang et al. [56] reported that an aqueous extract of *C. moschata*, rich in polysaccharides, was able to normalize GSH levels in rat macrophages exposed to the oxidizing agent H_2_O_2_, compared to cells not exposed to the oxidizing agent.

Figure 4F presents the results concerning the % of Caco-2 cell distribution through the cell cycle phases induced by AF and MF fractions of Pulp-HE, calculated from flow cytometry plots as those presented in Figure 4E. Firstly, none of the fractions tested significantly altered the percentage of cells in G0/G1 (*p* > 0.05). However, both fractions significantly increased the percentage of cells in S phase while simultaneously reducing G2/M cell population (*p* < 0.05). Thus, we observed that both fractions induce cell cycle arrest in S phase, preventing cell cycle progression to the G2/M phase. As ROS can induce DNA damage, and DNA damage is a major limitation for cell cycle progression at the various checkpoints, leading to cell cycle exit or cell death [45,46], it is likely that both oxidative stress and cell cycle arrest are correlated in cells exposed to Pulp HE-MF. In cells exposed to Pulp HE-AF, the fraction bioactive components can modulate cell cycle regulatory proteins, leading to cell cycle arrest and triggering cell death independent of oxidative stress.

Aristatile and Alshammari [20] reported that different extracts from *C. ficifolia* fruit pulp induce cell cycle arrest in the various phases in human bone marrow-mesenchymal stem cells (hBM-MSCs), depending on the solvent used for extraction (chloroform, hexane, or methanol) and on the concentration.

#### 2.5.2. Effect of Peel HE Extract Fractions

**Figure 5:**
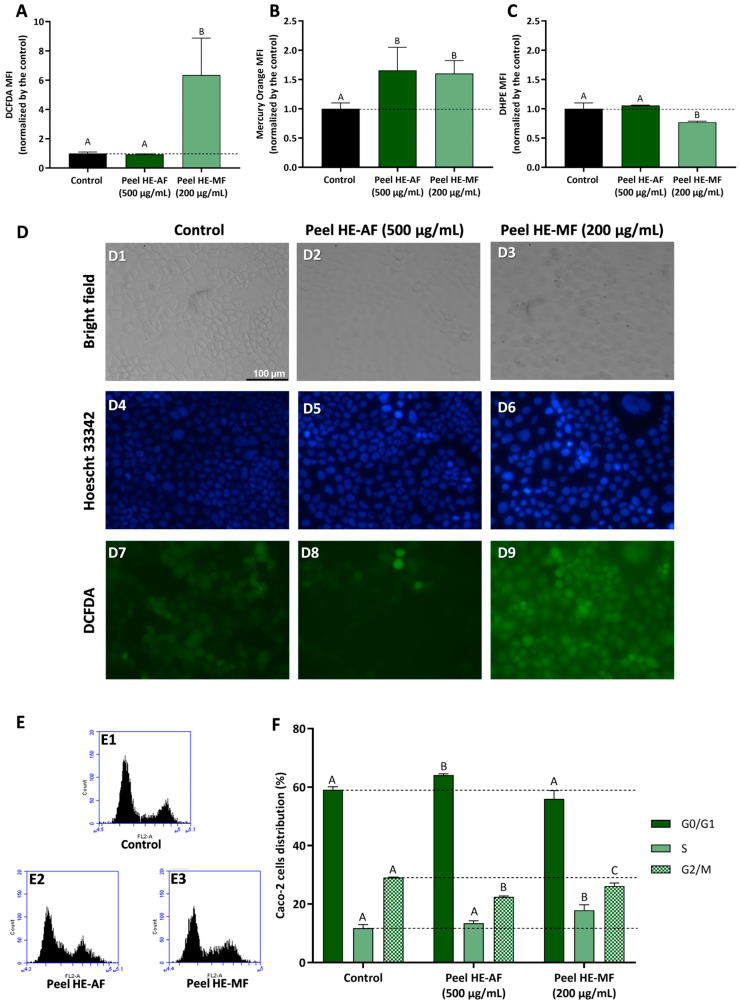
Effect of *C. ficifolia* fruit peel HE extract’s fractions (AF and MF) on oxidative stress markers and cell cycle arrest in Caco-2 cells exposed for 24 h to Peel HE-AF (500 µg/mL) and Peel HE-MF (200 µg/mL). (**A**–**C**): Intracellular ROS levels (evaluated as DCFDA MFI; panel (**A**)), glutathione content (GSH; evaluated as Mercury orange MFI, panel (**B**)), and lipidic peroxidation (evaluated as DHPE MFI, panel (**C**)). MFI: mean fluorescence intensity. (**D**): Assessment of Caco-2 cells morphology (bright-field), DNA damage integrity (Hoescht 33,342 probe; DAPI filter), and ROS levels (DCFDA; FITC filter); Control (D1, D4, and D7), exposure to AF (D2, D5, and D8), and exposure to MF (D3, D6, and D9); scale bar: 100 µm, magnification of 200×; (**E**,**F**): Assessment of fractions-induced cell cycle arrest induced (**F**) calculated for flow cytometry plots as exemplified in panel (**E**). Statistically different differences (*p* < 0.05) between fractions are denoted with different letters. Results are presented as mean ± SD.

Figure 5 presents the results concerning fractions-induced modulation of oxidative stress markers and cell cycle progression in Caco-2 cells treated with Peel HE-AF (500 µg/mL) and Peel HE-MF (200 µg/mL) for 24 h. Cells exposed to 500 µg/mL of Peel HE-AF present control-like levels of ROS and lipid peroxidation but present a significant increase (*p* < 0.05) in intracellular GSH (Figure 1, panels A–C), identical to the results obtained for Pulp HE-AF (Figure 3), and confirming that AF from both pulp and peel does not induce oxidative stress, which is in line with the low cytotoxicity of these fractions at 500 µg/mL.

Due to the higher cytotoxicity induced by Peel HE-MF (Figure 3), cells were exposed to 200 µg/mL, the lowest concentration that reduced cell viability. Also, this concentration reduced Caco-2 cell viability, likely the other fractions tested, which allows for a better results comparison. Peel HE-MF induced a significant increase (*p* < 0.05) in ROS and lipid peroxidation while also increasing GSH content (Figure 5). The pattern is like that obtained for Pulp HE-MF, although 500 µg/mL of Pulp HE-MF induced a ~2-fold increase in ROS levels (compared to non-exposed cells), while 200 µg/mL of Peel HE-MF induced a ~6-fold increase in ROS levels. Additionally, GSH content is identical in cells exposed to the two MF fractions, which indicates that Caco-2 cells’ endogenous antioxidant mechanisms are likely unable to cope with the oxidative damage induced by Peel HE-MF, as evidenced by the higher cytotoxicity observed (Figure 3), the higher number of cells with morphological changes (Figure 5, panel D3), and cells with loss of DNA integrity (Figure 5, panel D6). Fluorescence microscopy also confirmed the increased ROS content in cells exposed to Peel HE-MF. As observed for Pulp HE-AF (Figure 4), bright-field and fluorescence microscopy analysis show that cells exposed to Peel HE-AF overall present morphology, ROS levels, and DNA integrity identical to non-exposed cells (Figure 4, panels D2, D5, and D8).

Figure 5F presents the results concerning the % of Caco-2 cell distribution through the cell cycle phases, calculated from flow cytometry plots as exemplified in panel E. We observed that the percentage of cells in G0/G1 phase increases for cells exposed to Pell HE-AF when compared to control (*p* < 0.05), in addition to a slight increase of cells’ percentage in S phase ((*p* > 0.05) and a reduction in G2/M. Thus, 500 µg/mL of Pell HE-AF induces cell cycle arrest in the G0/G1 phase in Caco-2 cells. The effect of Pell HE-MF on cell cycle progression is like the effect observed for fractions obtained from the pulp, as the fraction induces cell cycle arrest in S phase, seen as an increase of the percentage of cells in this phase, accompanied by a decrease in G2/M (Figure 5F).

To the best of our knowledge, this is the first report on the effect of *Cucurbita* spp. fruit peel extracts on oxidative stress markers and cell cycle arrest in cell models. The peel is considered a by-product and is not typically consumed, and thus further studies should focus on the additional study of its anti-tumoral potential aiming at the valorization of this by-product aligned with zero-waste policies.

### 2.6. Pulp and Pell C. ficifolia HE Extracts Fractions Induce Anti-Inflammatory Activity

To evaluate the anti-inflammatory activity of *C. ficifolia* HE extracts’ fractions, the in vitro model of lipopolysaccharides (LPS)-stimulated RAW 264.7 cells were used since LPS activates inflammatory signaling pathways that culminate in the activation of inducible nitric oxide synthase (iNOS), which produces nitric oxide (NO). This signaling molecule diffuses to the extracellular space where it can be quantified using the Griess reagent. Thus, to evaluate the anti-inflammatory capacity of the different fractions (AF and MF) of the two extracts (Pulp HE and Peel HE), RAW 264.7 cells were exposed to the extract fractions (24 h, at non-cytotoxic concentrations; maximum 100 μg/mL, with cell viability above 90%), in the presence and absence of LPS, as described in methods.

**Figure 6:**
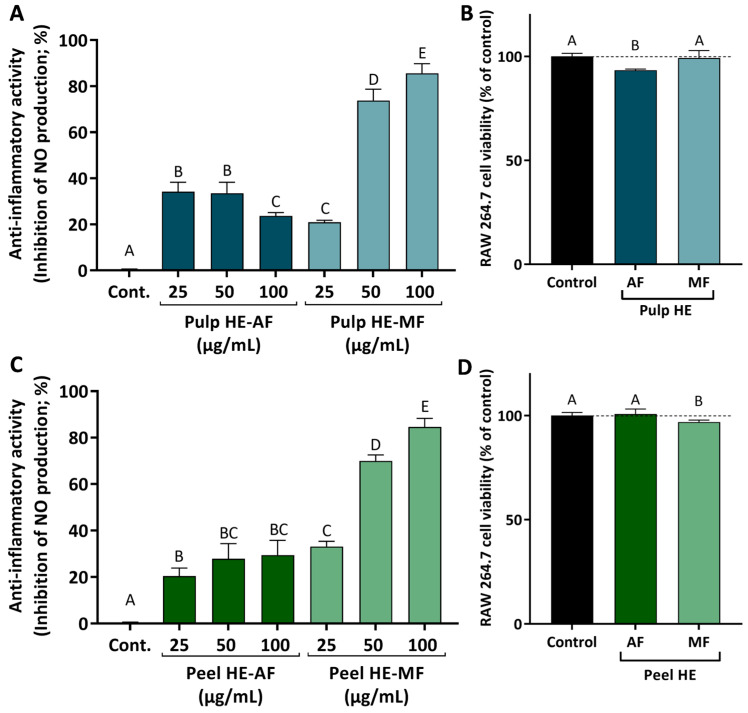
Anti-inflammatory activity induced by AF and MF obtained from *C. ficifolia* Pulp HE and Peel HE extracts (**A**,**C**; respectively), measured as the inhibition of nitric oxide (NO) release by LPS-stimulated RAW 264.7 cells, expressed as a percentage of control. Evaluation of the safety profile of the fractions, at 100 µg/mL, on RAW 264.7 cell viability (**B**,**D**; respectively). Statistically significant differences (*p* < 0.05) between fractions are denoted with different letters. Results are presented as mean ± SD.

Figure 6 presents the results obtained for the anti-inflammatory activity, evaluated as the ability of the HE extracts fraction to reduce NO production in LPS-stimulated RAW 264.7 cells. Firstly, the safety profile of the various fractions was assessed, and we observed that AF and MF from both pulp and peel induced no or low cytotoxicity in cells exposed for 24 h to 100 ug/mL (Figure 6B,D).

Comparing fraction types, MFs of both pulp and peel induced higher inhibition of NO production when compared to AFs. As observed, both MFs induce a dose-dependent inhibition of NO release, achieving a maximum inhibition of ~80% in LPS-stimulated cells exposed to 100 μg/mL of Pulp HE-MF or Peel HE-MF (Figure 6A,C).

For the effect of AFs, Peel HE-AF inhibited NO release in a dose-dependent pattern (average values), although differences are not statistically significant (*p* > 0.05). None of the AF concentrations tested (up to 100 μg/mL) was able to induce a higher inhibition than 25 μg/mL of Peel HE-MF (Figure 6C). Concerning the effect of Pulp HE-AF, a maximum inhibitory activity of 35% was observed for cells treated with 25 and 50 μg/mL Pulp HE-AF, being this the highest effect observed, as inhibition of NO release decreased in cells exposed to 100 μg/mL, likely due to the slight cytotoxicity observed at this concentration (Figure 6B), which may limit the anti-inflammatory activity. Thus, MF fractions present a dose-dependent anti-inflammatory activity (*p* < 0.05) and potential as anti-inflammatory agents, whose additional molecular mechanisms should be exploited in future studies. These results point to *C. ficifolia* as a functional food, and its consumption may contribute to reduction in symptoms of bowel inflammatory diseases or in their prevention. Indeed, recent studies demonstrated that a triterpene (cucurbitacin E) present in pumpkin exerts a protective effect by modulating inflammatory pathways, having therapeutic potential in colitis [57]. Other cucurbitacins, such as cucurbitacin B [58], have been proposed to have a wide range of pharmacological activities, including anti-inflammatory, neuroprotective, anti-diabetic, and anti-cancer [58,59].

In studies carried out using an animal model of obesity (monosodium glutamate-induced obesity in mice), it was found that consumption of *C. ficifolia* fruit aqueous extract (200 mg/kg/day) decreased the expression of pro-inflammatory mediators [tumor necrosis factor alpha (TNF-α), interleukin-6 (IL-6) in the adipose tissue and also increased the expression of the anti-inflammatory cytokine interleukin-10 (IL-10) in lean mice [21]. Similarly, in a streptozotocin-induced mouse model of diabetes, an aqueous extract of *C. ficifolia* fruit (200 mg/kg/day) reduced the mRNA expression of TNF-α and IL-6 in the liver [13]. *C. ficifolia* extracts contributed to an anti-inflammatory state in adipocytes, an effect that is attributed to its high content of D-chiro-inositol [17,60]. Recently, in a co-culture model of 3T3-L1 adipocytes and RAW-264.7 macrophages, an aqueous extract of *C. ficifolia* was able to suppress meta-inflammation, an effect that was mediated by their secretomes [61].

Considering other *Cucurbita* spp., an aqueous extract of *C. pepo* (100 mg/kg/day) reduced LPS-induced toxicity and inflammation in the brain of C57BL/6 mice [62]. To the best of our knowledge, this is the first report on the reduction of nitric oxide production induced by extracts of *C. ficifolia*.

## 3. Materials and Methods

### 3.1. Standards and Reagents

Ethanol (HPLC grade), methanol (HPLC grade), formic acid (HPLC grade), hydrochloric acid (HPLC grade), aluminum chloride (III), Folin–Ciocalteu’s reagent, sodium molybdate, gallic acid, catechin, 2,2′-azino-bis(3-ethylbenzothiazoline-6-sulfonic acid) diammonium salt (ABTS), potassium persulfate, (±)-6-hydroxy-2,5,7,8-tetramethylchromane-2-carboxylic acid (Trolox), *N*-1-naphthylethylenediamine dihydrochloride, sulfanilamide, salicylic acid, iron (II) sulphate, hydrogen peroxide (30% solution), rhamnose, arabinose, galactose, glucose, sulfuric acid (87% solution), 2-desoxiglucose, and lipopolysaccharides (LPS) were purchased from Sigma-Aldrich/Merck**^®^** (Algés, Portugal). Enzymes and reagents for enzymatic assays were purchased from Sigma-Aldrich/Merck (Algés, Portugal). Fetal bovine serum (FBS), L-glutamine, Dulbeco’s Modified Eagles Medium (DMEM), streptomycin, and penicillin were purchased from Gibco, Alfagene**^®^** (Lisboa, Portugal). Alamar Blue and Hoescht 33,342 were purchased from Invitrogen, Alfagene**^®^** (Lisboa, Portugal). DCFDA (2′,7′-dichlorofluorescein diacetate), DHPE-FITC [(fluorescein-5-thiocarbamoyl)-1,2-di-hexadecanoyl-*sn*-glycero-3 phosphoethanolamine], and Mercury orange (1-(4-chloromercuriophenylazo)-2-naphthol) were purchased from Thermo Fisher Scientific (Alfagene, Lisboa, Portugal). RNAse was obtained from NZYtech (Lisboa, Portugal).

### 3.2. Plant Material

Fresh fruits of *Cucurbita ficifolia* Bouché, acquired from local producers, were grown under organic farming conditions and harvested ripe in October 2017 on a farm. The fruit was rinsed with distilled water to remove any dirt, and then the pulp, peel, and seeds were separated. After weighing, the peel and pulp were frozen separately and lyophilized in a Dura Dry TM μP freeze-drier (−45 °C; 250 mTorr). After lyophilization, both peel and pulp were separately ground.

#### 3.2.1. Preparation of Extracts

The lyophilized ground peel and pulp samples were used to obtain hydroethanolic (HE) extraction, following the exhaustive extraction method described by Martins-Gomes et al. [28], with some modifications. Briefly, to 1 g of each lyophilized ground material, 50 mL of a hydroethanolic solution (80/20, % *v*/*v*) were added, followed by agitation of the mixture in an orbital shaker (1 h, 150 rpm; Orbital Shaker GFL 3005 series, Hannover, Germany) at room temperature. Afterwards, the mixture was centrifuged (7000 rpm, 4 °C) for 5 min (Sigma Centrifuges 3–30 K, St. Louis, MO, USA), the supernatant was collected, and the pellet was used to repeat the extraction process described above, for a total of four times. The four supernatants were combined and filtered using a glass fiber filter (1.2 µm glass fiber filters, Grade 693; VWR International Ltd., Carnaxide, Portugal), followed by concentration and removal of ethanol using a rotary evaporator (35 °C). The extracts were then frozen, lyophilized, weighed for yield calculation, and properly stored until further use.

#### 3.2.2. Fractionation of Extracts

Pulp and peel HE extracts obtained in Section 3.2.1. were fractionated by solid-phase extraction (SPE) using C18 SPE cartridges (20 mL; 5 g packing; Supelclean LC-18 SUPELCO, Sigma-Aldrich/Merck, Germany). Before fractionation, the column’s stationary phase was activated with 120 mL of methanol and then conditioned with 120 mL of ultrapure distilled water. Pulp and peel HE extracts (1 g) were dissolved in 140 mL of ultrapure distilled water, and the pH of the extracts was adjusted to pH = 2 using 1 M HCl solution, followed by centrifugation (7000 rpm, 4 °C; 5 min). The supernatants were then collected, filtered (1.2 µm glass fiber filters, Grade 693, VWR International Ltd.), and applied in the SPE cartridge. Fractionation was started with the elution of water-soluble components, using 125 mL of ultrapure distilled water, resulting in the aqueous fraction (AF). Then, methanol-soluble compounds were eluted using 125 mL of methanol, producing the methanolic fraction (MF). The procedure was performed separately for peel and pulp extracts. Each fraction was then concentrated using a rotary evaporator (35 °C), a step that also allowed for the removal of all methanol in the MF. All fractions were frozen, lyophilized, and stored until analysis. AF and MF fractions obtained from pulp HE will be referred to as Pulp HE-AF and Pulp HE-MF. AF and MF fractions obtained from peel HE will be referred to as Peel HE-AF and Peel HE-MF, respectively.

### 3.3. Total Phenolic, Total Flavonoids, and Ortho-Diphenols Content

The total phenol content (TPC), *ortho*-diphenol content (ODC), and total flavonoid content (TFC) of pulp and peel fractions were determined using colorimetric methods, namely the Folin–Ciocalteau reagent method (TPC), aluminum complexation (TFC), and molybdenum complexation (ODP). All methodologies were performed in 96-well microplates, using a method adapted from [63]. All absorbance measurements were performed using a microwell plate reader (Multiskan SkyHigh; ThermoFisher Scientific, Waltham, MA, USA). TPC and ODC were expressed as gallic acid equivalents (mg GA eq/g extract), and TFC was expressed as catechin equivalents (mg C eq/g extract).

### 3.4. In Vitro Antioxidant Activity Assessment

#### 3.4.1. ABTS Radical Scavenging Assay

ABTS [2,2′-azino-bis(3-ethylbenzothiazoline-6-sulphonic acid)] radical scavenging was assessed as described by Singleton et al. [64], adapted for 96-well microplates. ABTS^•+^ radical was produced by mixing 7.4 mM of ABTS with 2.45 mM of potassium persulfate (K_2_S_2_O_8_) in a ratio of 1:1 (*v*/*v*). The mixture was allowed to react for 15–16 h in the dark at room temperature. Then, the radical solution was diluted in methanol to obtain a solution with an absorbance of 0.70 ± 0.02 at a wavelength of 734 nm. To 190 µL of ABTS^•+^ solution, 10 μL of each sample (1 mg/mL) were added. Absorbance was read at 734 nm after 6 min of incubation at room temperature (in the dark). A standard curve was prepared using 6-hydroxy-2,5,7,8-tetramethylchroman-2-carboxylic acid (Trolox; 0–0.5 mM). The results were expressed as Trolox equivalents (mmol Trolox eq/g extract, as indicated in the results).

#### 3.4.2. Hydroxyl Radicals Scavenging Assay

The hydroxyl (^•^OH) radical scavenging capacity was performed as described by Halliwell [65], adapted to 96-well microplates. To 50 μL of test solution (ranging from 0.03125 to 0.50 mg/mL), 50 µL of FeSO_4_ solution (6 mM) and 50 µL of hydrogen peroxide (H_2_O_2_) solution (6 mM) were added. The mixture was homogenized using a vortex mixer and incubated for 10 min at room temperature. Then, 50 µL of salicylic acid (6 mM; prepared in ethanol) was added to the mixture. After incubation for 30 min (room temperature), absorbance was measured at a wavelength of 510 nm against a blank containing the above mixture without the sample. The percentage decrease of absorbance against a blank sample was calculated using Equation (1):

(1) $$Inhibition\;(\%)=\frac{\left(Blank\;abs\;510-Sample\;abs\;510\right)}{\left(Blank\;abs\;510\right)}\;\times \;100$$

### 3.5. Profiling and Quantification of Individual Phenolic Compounds by HPLC-DAD and HPLC-DAD-ESI-MS^n^

Identification and quantification of individual phenolic compounds in AF and MF of pulp and peel HE extracts were performed using high-performance liquid chromatography (HPLC), coupled to a photodiode array detector (DAD) for compound quantification and mass spectrometry detector for compound identification.

The quantification of phenolic compounds was performed using an Ultimate 3000 HPLC system (Dionex, USA), equipped with an Ultimate 3000 pump, an Ultimate 3000 column compartment, a WPS-3000 TSL Analyt auto-sampler, and a PDA-100 photodiode array detector. For chromatographic separation, a C18 column was used (Ace 5, particle size 5 μm, 250 mm × 4.6 mm; Advanced Chromatography Technologies; Aberdeen, Scotland). A 5% formic acid solution (prepared in ultra-pure distilled water; solvent A) and methanol (HPLC grade; solvent B) were used as eluents, and the elution program, temperature, and detection parameters were performed as described by [28]. The fractions were injected at 10 mg/mL, and 100 μL of each sample were injected in each run. For analysis, data acquisition, and peak integration, the Chromeleon (Version 7.3; Dionex, Sunnyvale, CA, USA) was used. The quantification was performed using standard curves of the phenolic compounds with higher structural similarity, namely, luteolin derivatives were quantified as luteolin-7-*O*-glucoside; quercetin and rhamnetin derivatives were quantified as quercetin-3-*O*-glucoside.

Phenolic compound identification by HPLC-DAD-ESI-MS^n^ was achieved using a Thermo Scientific Ultimate 3000 system equipped with an auto-sampler, pump, and photodiode array detector, and an LTQ XL Linear trap detector. Chromatographic separation was performed using a Luna C18 column (250 × 4.6 mm, 5 μm particle size); the solvents were at 0.1% formic acid (prepared in ultra-pure distilled water; solvent A) and methanol (LC-MS grade; solvent B). Mass spectrometer ionization was performed by electrospray ionization (ESI) in negative mode. Elution program, temperature, injection conditions, capillary conditions, and detection parameters were performed as described by [28]. Identification of individual compounds was performed based on UV-VIS spectra, fragmentation patterns, and comparison to the literature [35,36].

### 3.6. Determination of Sugars Content by HPAEC-PAD

For the determination of rhamnose, arabinose, galactose, and glucose content in the AFs and MFs of pulp and peel HE extracts, each fraction was subjected to acid hydrolysis. To 5 mg of each fraction, 400 µL of sulfuric acid solution (72%) were added, followed by a 3 h incubation at room temperature, with homogenization of the mixture every 30 min. Then, 4 mL of ultra-pure water were added, and the mixture was incubated in a thermoblock for 2.5 h at 100 °C, after which 500 µL of internal standard (2-desoxiglucose at 1 mg/mL) were added. Then, the samples were centrifuged (5 min, 500× *g*), the supernatant was collected, and analyzed by high performance anion exchange chromatography with pulsed amperometric detection (HPAEC-PAD, ICS-3000, Dionex) using a CarboPac PA-20 column [66]. The injection and detector parameters, eluents, and elution program were performed as described by Vilela et al. [66].

### 3.7. Assessment of In Vitro Enzyme Inhibition Capacity

The anti-aging, neuroprotective, and anti-diabetic potential of AF and MF of *C. ficifolia* fruit pulp and peel HE were evaluated using in vitro enzymatic inhibition assays, as described by Taghouti et al. [63]. The assessment of anti-aging potential was carried out through the ability to inhibit the enzymes elastase and tyrosinase. Neuroprotection was evaluated as the anti-acetylcholinesterase (AChE) and anti-tyrosinase activity. The α-amylase and α-glucosidase inhibition was used to evaluate the anti-diabetic activity. All extract fractions were tested at 300 µg/mL. Stock solutions of the fractions were prepared in DMSO at 20 mg/mL. The final DMSO concentration never exceeded 2.5% DMSO, which was previously shown to have no enzymatic inhibitory effect [67].

### 3.8. Cell Culture Maintenance and Cytotoxic/Anti-Proliferative Activity Assessment

In order to evaluate the safety profile and anti-proliferative activity of AF and MF fractions obtained from *C. ficifolia* pulp and peel HE extracts, stock solutions were prepared in DMSO at 20 mg/mL. The final DMSO concentration in the test solutions never exceeded 2%. The cell lines used were HepG2 (human hepatocellular carcinoma; ATCC, Rockville, MD, USA), Caco-2 (human colon adenocarcinoma; Cell Line Service (CLS), Eppelheim, Germany), RAW 264.7 (mouse macrophages, from Abelson murine leukaemia virus-induced tumor; CLS, Eppelheim, Germany), and HaCaT (human keratinocytes, CLS, Germany [68]). Cell handling, maintenance, and seeding were performed as described by Silva et al. [69].

To test the effect of extract fractions on cell viability, cells were seeded in 96-well culture microplates (5 × 10^4^ cells/mL, 100 µL/well) and allowed to adhere and stabilize for 48 h. After this period, the culture medium was removed, and 100 µL of test solutions, prepared in FBS-free culture medium, were added to each well. AF and MF fractions of both HE extracts were tested at concentrations between 100 and 750 μg/mL for 24 or 48 h (in independent assays). After incubation, test solutions were removed and replaced with 100 µL of 10% (*v*/*v*) Alamar Blue solution (diluted in FBS-free culture media). After 5 h of incubation, absorbance was measured at 570 and 620 nm in a microplate reader (Multiskan EX, MTX Labsystems, Inc. Bradenton, FL, USA). Cell viability (% of control normalized by non-exposed cells) was subsequently calculated, as previously described [69].

### 3.9. Anti-Inflammatory Activity

To evaluate the anti-inflammatory properties of the different pulp and peel extract fractions, the LPs-stimulated RAW 264.7 cell model was used as described by Silva et al. [69]. RAW 264.7 cells were seeded in 96-well microplates in the conditions described in Section 3.8. Cells were then exposed to test solutions (25 to 100 μg/mL) in the presence and absence of LPS (1 μg/mL) for 24 h. After this incubation, 50 μL of supernatant from each well was transferred to a new 96-well microplate, to which 50 μL of Griess reagent [equal volumes of 0.1% (*w*/*v*) *N*-(1-naphthyl) ethylenediamine dihydrochloride prepared in distilled water and 1% (*w*/*v*) sulfanilamide prepared in 5% (*w*/*v*) H_3_PO_4_ (*v*/*v*)] were added. After agitation, the mixture was incubated for 10 min (in the dark, at room temperature), and then the absorbance was measured at 540 nm in a Multiskan EX microplate reader (Thermo Scientific, Porto, Portugal). Nitric oxide was quantified using a NaNO_2_ calibration curve (0–100 μM), and the results are expressed as a percentage of nitrite production (normalized to LPS-stimulated cells, set at 100%).

### 3.10. Assessment of DNA Fragmentation and Intracellular ROS Content Using Fluorescence Microscopy

The effect of AF and MF of *C. ficifolia* peel and pulp HE extracts on DNA fragmentation and intracellular ROS content was evaluated in Caco-2 cells seeded in 12-well microplates (5 × 10^4^ cells/mL, 750 µL/well). Cells were exposed to different fractions (Pulp HE-AF, Pulp HE-MF, and Peel HE-AF at 500 μg/mL; Peel HE-MF at 200 μg/mL) for 24 h. After incubation, test solutions were removed, and the cells were washed with PBS. Then, Caco-2 cells were treated with 500 μL of 20 μM DCFDA (2′,7′-dichlorofluorescein diacetate) solution, prepared in FBS-free DMEM, followed by 45 min incubation (37 °C). After incubation, probe solution was removed, cells were washed once with PBS, and then 500 μL of PBS containing 5 μg/mL of Hoechst 33,342 probes (Invitrogen, Alfagene Portugal) were added [70]. After 5 min incubation (room temperature, in the dark), cells were observed under a fluorescence microscope (Olympus IX51), equipped with DAPI and FITC filters. Image acquisition was performed using a CCD camera and Cell A^ image acquisition software.

### 3.11. Assessment of Intracellular ROS Content, GSH Content, Lipid Peroxidation, and Cell Cycle Arrest Using Flow Cytometry

Flow cytometry evaluation of oxidative stress markers and cell cycle arrest was performed using one-color or double-color assays. Flow cytometry data acquisition was performed using a BD Accuri™ C6 flow cytometer (Becton Dickinson, CA, USA). In each assay, 10,000 events were acquired per sample. Data analysis was performed using BD Accuri™ C6 software, version 1.0.264.21 (Becton Dickinson, CA, USA).

Caco-2 cells, seeded in 12-well microplates (5 × 10^4^ cells/mL, 750 µL/well), were exposed to different extract fractions (Pulp HE-AF, Pulp HE-MF, and Peel HE-AF at 500 μg/mL; Peel HE-MF at 200 μg/mL) for 24 h. After this period, the cells were washed and detached using trypsin-EDTA solution, transferred to microtubes, and centrifuged (benchtop mini centrifuge, 5 min, at 500× *g*). Then, the supernatants were discarded, cells were washed once with PBS, and centrifuged. The supernatant was discarded, and the cells were resuspended in 600 µL of PBS. From each sample, 200 µL were transferred to three microtubes, comprising three identical sample sets, used to individually evaluate intracellular ROS, glutathione (GSH) content, and lipid peroxidation.

Intracellular ROS was assessed using the DCFDA probe (Invitrogene, Alfagene, Portugal); GSH levels were evaluated using the Mercury orange ([1-(4-chloromercuriophenyl-azo-2-naphthol)], Sigma-Aldrich/Merck, Germany) probe, and lipid peroxidation was evaluated using the DHPE-FITC [(fluorescein-5-thiocarbamoyl)-1,2-di-hexadecanoyl-*sn*-glycero-3 phosphoethanolamine] probe. All assays were performed as described by Silva et al. [71].

To access cell-cycle arrest, Caco-2 cells were treated as described above for oxidative stress markers evaluation, but after trypsinization and removal of the trypsin-EDTA solution by centrifugation, the cells were resuspended with ice-cold PBS (~4 °C). Then, cells were centrifuged, the supernatant discarded, and 500 μL of EtOH:PBS solution (70:30 *v*/*v*; previously cooled at −20 °C) was added for cell fixation. Samples were then stored at −20 °C for at least 4 h. After fixation, the cells were centrifuged, and the supernatant was discarded. Cells were washed twice with PBS, and then 200 μL of DNA staining solution (50 μg/mL of propidium iodide, 50 μg/mL of RNAse A, and 0.1% Triton X-100, diluted in PBS) were added to each sample. After 30 min incubation (37 °C, in the dark), cells were washed once with PBS, resuspended in PBS, and the events were acquired by flow cytometry [70].

### 3.12. Data and Statistical Analysis

The results are presented as mean ± standard deviation (SD). At least three independent assays were performed for each experiment. The IC_50_ (concentration that inhibits 50% of cell viability/proliferation) values were calculated from dose-response curves using Excel or GraphPad Prism tools. For each exposure time (24 and 48 h) and for each cell line, 3 independent assays were done (each one in quadruplicate). For each condition, IC_50_ values are expressed as mean ± SD of IC_50_ values calculated for the 3 independent experiments. Data and statistical analysis, as well as graphical design, were performed using GraphPad Prism (Version 8; GraphPad Software Inc., San Diego, CA, USA) and Microsoft Office Excel (Microsoft Corporation, Washington, DC, USA). One-way (single comparison) and two-way (multiple comparison) analyses of variance (ANOVA), followed by Tukey’s multiple test (significance level of 0.05), were applied.

## 4. Conclusions

In this work we describe for the first time the fractioning of hydroethanolic extracts obtained from pulp and peel of *C. ficifolia*, their chemical characterization, and bioactivities assessed using several methods, aiming at valorization of this fruit as functional food as well as the valorization of its by-products. Regarding the chemical characterization, all fractions are rich in carbohydrates, and only the MF fraction of peel HE contained quantifiable amounts of glycoside derivatives of quercetin, luteolin, and (iso)-rhamnetin. It was found that the Pulp HE-AF and Peel HE-MF are the ones with the highest content in sugars (after acidic hydrolysis). All fractions present antioxidant activity, being able to scavenge both ABTS^•+^ and ^•^OH radicals.

Regarding the biological activities of the fractions, it was also found that both extracts show anti-diabetic, neuroprotective, and anti-aging activities, with these activities being slightly higher for both MFs. Pulp HE fractions have no/low toxicity against Caco-2, HepG2, and HaCaT cells, and AF has a protective effect since it increases intracellular GSH content. All fractions show a slight potential to arrest the cell cycle in G0/G1 or S phases, depending on the fraction. All fractions induced anti-inflammatory activity, with MF being more effective in reducing LPS-induced NO production in RAW 264.7 cells. Both MF induces an increase in ROS content and a slight increase in lipid peroxidation. However, all fractions increased intracellular GSH content. We therefore conclude that the pulp can be considered a functional food due to the various health-promoting activities here described, while the peel extracts have potential as a source of molecules with anti-tumor activity for the development of pharmaceutical formulations.

## Figures and Tables

**Table 1:** *C. ficifolia* fruit pulp and peel extraction and fractionation yields. Total phenolic content, *ortho*-diphenol content, and flavonoid content of each extract and fraction, and evaluation of fractions’ antioxidant scavenging activity.

		Pulp Hydro-Ethanolic (Pulp HE)		Peel Hydro-Ethanolic (Peel HE)	
**Extraction yield** (%, *w*/*w*)		59.91 ± 5.22		28.66 ± 1.03	*
	**Fraction**				
**Fractionation yield** (%, *w*/*w*)	**AF**	93.26 ± 4.69	*	99.26 ± 0.55	*
	**MF**	2.91 ± 0.44		6.08 ± 1.60	
		**Chemical composition**			
**Total phenols** (mg GA eq/g extract)	**AF**	51.40 ± 0.36	n.s.	52.66 ± 2.30	*
	**MF**	58.15 ± 4.65		99.05 ± 1.51	
***Ortho*-diphenol** (mg GA eq/g extract)	**AF**	252.19 ± 17.83	n.s.	232.73 ± 3.59	*
	**MF**	257.55 ± 15.84		398.88 ± 4.96	
**Total Flavonoids** (mg CAT eq/g extract)	**AF**	115.34 ± 6.28	*	105.62 ± 4.07	*
	**MF**	132.60 ± 7.64		143.29 ± 7.18	
		**Antioxidant activity**			
**ABTS^•+^** (mmol Trolox eq./g extract)	**AF**	0.07 ± 0.01	*	0.19 ± 0.01	*
	**MF**	0.25 ± 0.02		0.66 ± 0.05	
**OH** (% inhibition; 0.5 mg/mL)	**AF**	33.66 ± 0.88	*	28.40 ± 0.37	*
	**MF**	44.88 ± 1.52		44.83 ± 1.12	

Notes: AF: aqueous fraction and MF: methanolic fraction; significant statistical differences between fractions (*) if *p* < 0.05. n.s. denotes non-significant. Results are presented as mean ± standard deviation (*n* = 3).

**Table 2:** Phytochemical composition of the different aqueous (AF) and methanolic (MF) fractions of hydroethanolic extracts (Pulp HE AF and MF and Peel HE AF and MF), determined by HPLC-DAD-ESI/MS^n^ (respective chromatograms are shown in Supplementary Material Figure S1).

Compound		R.T. (min)	ESI-MS^2^	Quantification (mg/g of Extract)			
				Pulp HE Extract		Peel HE Extract	
				AF	MF	AF	MF
1	(Iso)rhamnetin-(?)-*O*-deoxy-hexose-hexose-(?)-*O*-deoxy-hexose	32.84 ± 0.05	[769]:623;315	n.d.	n.d.	n.d.	0.52 ± 0.10 *
2	Quercetin-(?)-*O*-deoxy-hexose-hexose	33.59 ± 0.07	[609]:301	n.d.	n.d.	n.d.	0.48 ± 0.04 *
3	Luteolin-(?)-*O*-deoxy-hexose-hexose	38.40 ± 0.06	[593]:285	n.d.	n.d.	n.d.	0.20 ± 0.04 *
4	(Iso)rhamnetin-(?)-*O*-deoxy-hexose-hexose	39.98 ± 0.09	[623]:315;300	n.d.	n.d.	n.d.	2.73 ± 0.49 *
		Total phenolics		n.d.	n.d.	n.d.	3.93 ± 0.68 *

Notes: R.T.: retention time; ESI-MS^2^: Fragment ions obtained after fragmentation of the pseudo-molecular ion [M]^−^; n.d.: not detected; AF: aqueous fraction and MF: methanolic fraction; significant statistical differences between fractions (*) if *p* < 0.05. Results are presented as content in mg/g of extract as mean ± standard deviation (*n* = 3).

**Table 3:** Sugar content after acid hydrolysis of the different aqueous and methanolic fractions of hydroethanolic extracts, determined by HPAED-PAD.

	Compound	R.T. (min)	Quantification (mg/g of Extract)			
			Pulp HE Extract		Peel HE Extract	
			AF	MF	AF	MF
1	Rhamnose	7.57 ± 0.29	1.37 ± 0.22 ^a^	5.65 ± 0.41 ^b^	0.41 ± 0.14 ^c^	23.62 ± 0.90 ^d^
2	Arabinose	8.29 ± 0.26	1.15 ± 0.10 ^a^	4.82 ± 0.28 ^b^	0.76 ± 0.13 ^c^	5.31 ± 0.82 ^b^
3	Galactose	10.11 ± 0.31	12.28 ± 1.36 ^a^	10.44 ± 0.31 ^a^	27.45 ± 3.36 ^b^	26.75 ± 2.72 ^b^
4	Glucose	10.99 ± 0.40	239.49 ± 16.97 ^a^	91.42 ± 8.75 ^b^	146.84 ± 25.61 ^c^	173.73 ± 16.12 ^c^
	Total sugars		253.83 ± 17.92 ^a^	112.32 ± 9.70 ^b^	175.45 ± 29.02 ^c^	229.41 ± 17.88 ^ac^

Notes: R.T.: retention time; AF: aqueous fraction; MF: methanolic fraction; significant statistical differences between fractions for the same compound are denoted with different letters when *p* < 0.05. Results are presented as content in mg/g of dry extract as mean ± standard deviation (*n* = 3).

**Table 4:** Evaluation of the neuroprotective, anti-aging, and antidiabetic potential of AF and MF fractions of *C. ficifolia* (from pulp and peel HE extracts) through in vitro anti-enzymatic activity inhibition assays.

	Enzymatic Inhibition (%)					
	Pulp HE			Peel HE		
Enzyme	AF	MF		AF	MF	
AChE	23.31 ± 2.79	22.68 ± 0.01		21.47 ± 5.85	22.56 ± 1.73	
Elastase	100.00 ± 5.58	100.00 ± 13.67		72.37 ± 5.58	96.05 ± 5.58	*
α-Amylase	13.53 ± 0.74	34.65 ± 1.47	*	12.59 ± 0.45	24.09 ± 1.12	*
α-Glucosidase	n.d.	n.d.		n.d.	n.d.	
Tyrosinase	7.85 ± 0.95	12.11 ± 1.55	*	5.83 ± 1.9	8.52 ± 0.01	

Abbreviations: AChE, acetylcholinesterase; AF, aqueous fraction; MF, methanolic fraction; n.d., not detected. Samples tested at 300 µg/mL. Significant statistical differences between fractions (*) if *p* < 0.05. Results are presented as % of inhibition as mean ± standard deviation (*n* = 3).

## Data Availability

Data are contained within the article.

## Supplementary Materials

The following supporting information can be downloaded at: https://www.mdpi.com/article/10.3390/molecules30030557/s1, Figure S1. Mass spectrum of compounds identified in *Cucurbita ficifolia* Peel HE MF (HE MF: methanolic fraction of hydroethanolic extract), as indicated in Table 2. (A): (Iso)rhamnetin-(?)-*O*-deoxy-hexose-hexose-(?)-*O*-deoxy-hexose; (B): Quercetin-(?)-*O*-deoxy-hexose-hexose; (C): Luteolin-(?)-*O*-deoxy-hexose-hexose; (D): (Iso)rhamnetin-(?)-*O*-deoxy-hexose-hexose. References are cited in [28,35,36].
