# Supplementary material for: Anti-Inflammatory, Anti-Hyperglycemic, and Anti-Aging Activities of Aqueous and Methanolic Fractions Obtained from Cucurbita ficifolia Bouché Fruit Pulp and Peel Extracts
Source: Molecules. 2025 Jan 26;30(3):557. doi: 10.3390/molecules30030557 (PMC11819910; doi:10.3390/molecules30030557)
Supplement: Supplementary file 1 [file molecules-30-00557-s001.zip › molecules-3412689-supplementary.pdf]

molecules

MDPI

Supplementary material

Anti-Inflammatory, Anti-Hyperglycemic, and Anti-Aging Activities of Aqueous and Methanolic Fractions Obtained from *Cucurbita ficifolia* Bouché Fruit Pulp and Peel Extracts

Tiago E. Coutinho [1], Carlos Martins-Gomes [1,2], Liliana Machado-Carvalho [1,3], Fernando M. Nunes [2,4] and Amélia M. Silva [1,3,5,*]

1 Centre for Research and Technology of Agro-Environmental and Biological Sciences (CITAB), Cell Biology and Biochemistry Laboratory, University of Trás-os-Montes and Alto Douro (UTAD), Quinta de Prados, 5000-801 Vila Real, Portugal; tecoutinho@utad.pt (T.E.C.); camgomes@utad.pt (C.M.-G.); lilianac@utad.pt (L.M.-C.)
2 Chemistry Research Centre-Vila Real (CQ-VR), Food and Wine Chemistry Laboratory, University of Trás-os-Montes and Alto Douro (UTAD), Quinta de Prados, 5000-801 Vila Real, Portugal; fnunes@utad.pt
3 Department of Biology and Environment, School of Life Sciences and Environment, University of Trás-os-Montes and Alto Douro (UTAD), 5000-801 Vila Real, Portugal
4 Department of Chemistry, School of Life Sciences and Environment, University of Trás-os-Montes and Alto Douro (UTAD), 5000-801 Vila Real, Portugal
5 Institute for Innovation, Capacity Building and Sustainability of Agri-Food Production (Inov4gro), University of Trás-os-Montes and Alto Douro (UTAD), Quinta de Prados, 5000-801 Vila Real, Portugal
* Correspondence: amsilva@utad.pt; Tel.: +351-259-350-921

Figure S1. Mass spectrum of compounds identified in *Cucurbita ficifolia* Peel HE MF (HE MF: methanolic fraction of hydroethanolic extract), as indicated in Table 2. (**A**): (Iso)rhamnetin-(?)-*O*-deoxy-hexose-hexose-(?)-*O*-deoxy-hexose; (**B**): Quercetin-(?)-*O*-deoxy-hexose-hexose; (**C**): Luteolin-(?)-*O*-deoxy-hexose-hexose; (**D**): (Iso)rhamnetin-(?)-*O*-deoxy-hexose-hexose.

HPLC-DAD-ESI-MS^n chromatograms

Cucurbita ficifolia phenolic compound were identified by HPLC-DAD-ESI-MS^n using a Thermo Scientific Ultimate 3000 system equipped with an auto-sampler, pump and photodiode array detector, and LTQ XL Linear trap detector. Chromatographic separation was performed using a Luna C18 column (250×4.6 mm, 5 μm particle size), the solvents were: 0.1% formic acid (prepared in ultra-pure distilled water; solvent A) and methanol (LC-MS grade; solvent B). Mass spectrometer ionization was performed by electrospray ionization (ESI) in negative mode. Elution program, temperature, injection conditions, capillary conditions and detection parameters were performed as described by [1]. Identification of individual compounds was performed based on UV-VIS spectra, fragmentation pattern, and comparison to the literature [2, 3].

Figure 1S shows the HPLC-DAD-ESI-MS^n chromatograms of compounds identified in Peel HE MF (as indicated in Table 2).

A

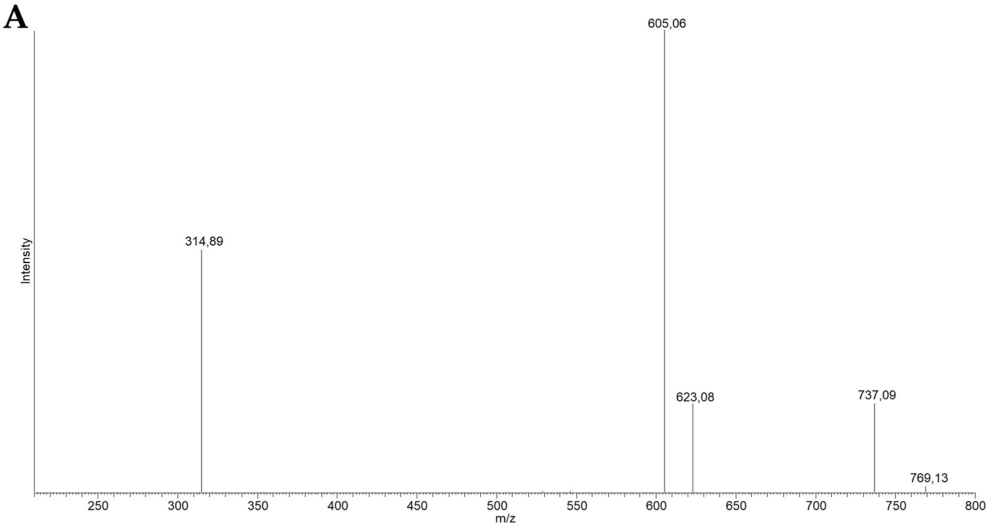

B

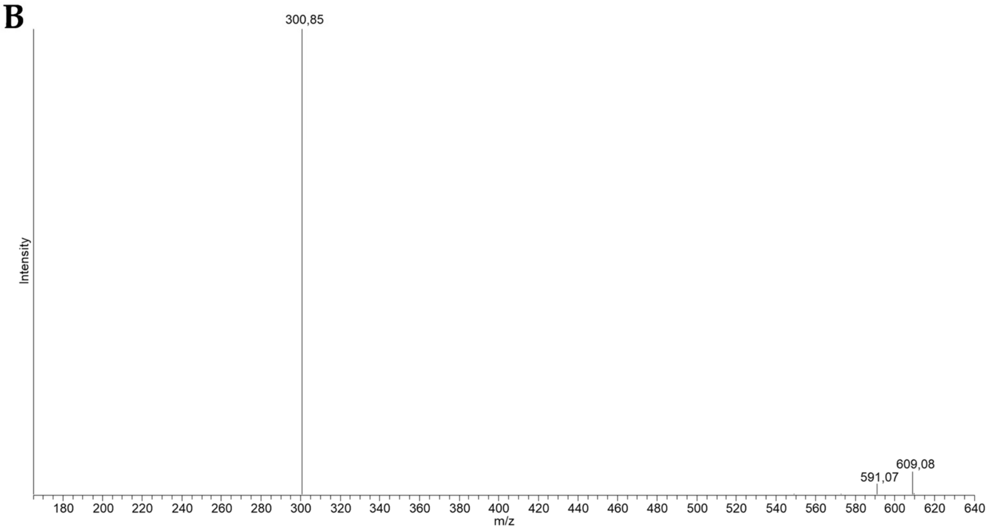

C

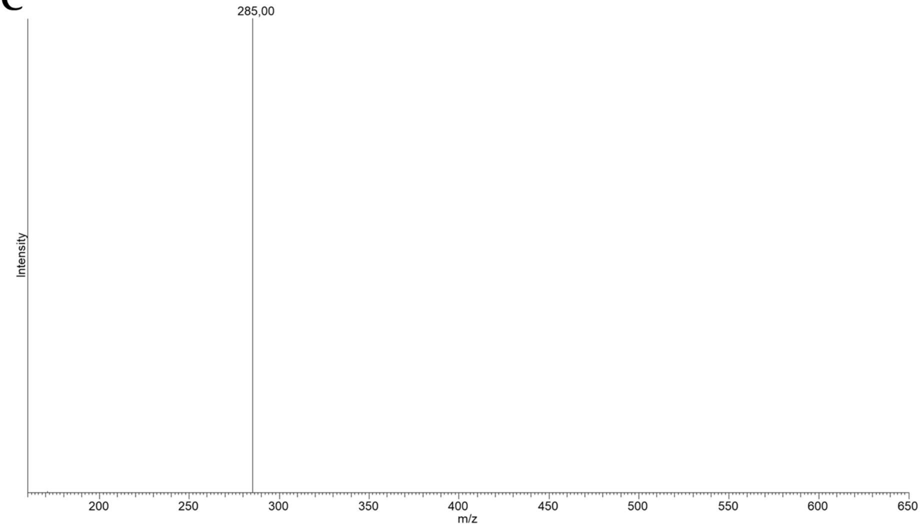

D

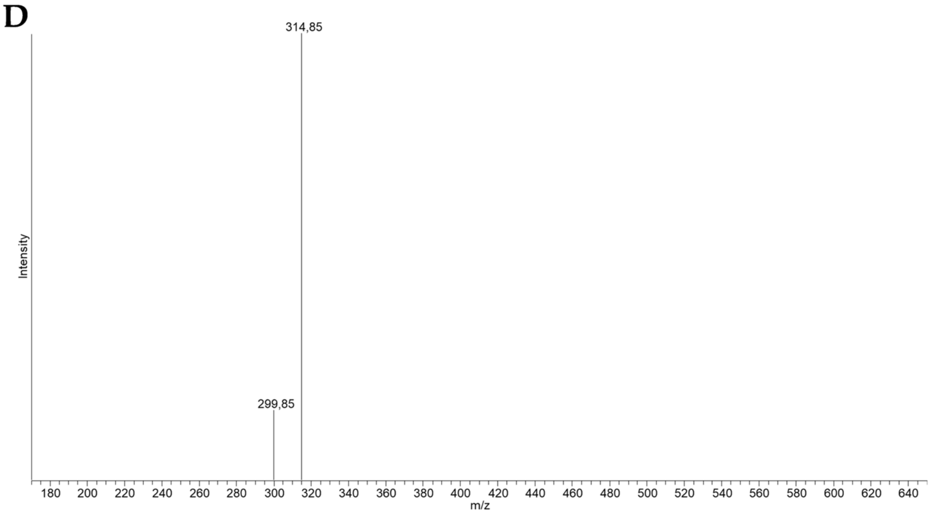

Figure S1. Mass spectrum of compounds identified in *Cucurbita ficifolia* Peel HE MF (HE MF: methanolic fraction of hydroethanolic extract), as indicated in Table 2. (**A**): (Iso)rhamnetin-(?)-*O*-deoxy-hexose-hexose-(?)-*O*-deoxy-hexose; (**B**): Quercetin-(?)-*O*-deoxy-hexose-hexose; (**C**): Luteolin-(?)-*O*-deoxy-hexose-hexose; (**D**): (Iso)rhamnetin-(?)-*O*-deoxy-hexose-hexose.

References

1. Martins-Gomes, Carlos, Meriem Taghouti, Judith Schäfer, Mirko Bunzel, Amélia M. Silva, and Fernando M. Nunes. "Chemical characterization and bioactive properties of decoctions and hydroethanolic extracts of Thymus carnosus Boiss." *Journal of Functional Foods* 43 (2018): 154-64.
2. Simirgiotis, M. J., and G. Schmeda-Hirschmann. "Direct identification of phenolic constituents in Boldo Folium (Peumus boldus Mol.) infusions by high-performance liquid chromatography with diode array detection and electrospray ionization tandem mass spectrometry." *Journal of Chromatography A* 1217, no. 4 (2010): 443-49.
3. Ding, S., E. Dudley, S. Plummer, J. Tang, R. P. Newton, and A. G. Brenton. "Fingerprint profile of Ginkgo biloba nutritional supplements by LC/ESI-MS/MS." *Phytochemistry* 69, no. 7 (2008): 1555-64.

Disclaimer/Publisher's Note: The statements, opinions and data contained in all publications are solely those of the individual author(s) and contributor(s) and not of MDPI and/or the editor(s). MDPI and/or the editor(s) disclaim responsibility for any injury to people or property resulting from any ideas, methods, instructions or products referred to in the content.
